# Frequent detection but lack of infectivity of SARS-CoV-2 RNA in presymptomatic, infected blood donor plasma

**DOI:** 10.1172/JCI159876

**Published:** 2022-09-01

**Authors:** Paula Saá, Rebecca V. Fink, Sonia Bakkour, Jing Jin, Graham Simmons, Marcus O. Muench, Hina Dawar, Clara Di Germanio, Alvin J. Hui, David J. Wright, David E. Krysztof, Steven H. Kleinman, Angela Cheung, Theresa Nester, Debra A. Kessler, Rebecca L. Townsend, Bryan R. Spencer, Hany Kamel, Jacquelyn M. Vannoy, Honey Dave, Michael P. Busch, Susan L. Stramer, Mars Stone, Rachael P. Jackman, Philip J. Norris, for the NHLBI Recipient Epidemiology and Donor Evaluation Study-IV-Pediatric (REDS-IV-P)

**Affiliations:** 1 Scientific Affairs, American Red Cross, Gaithersburg, Maryland, USA.; 2 Westat, Rockville, Maryland, USA.; 3 Vitalant Research Institute, San Francisco, California, USA.; 4 Department of Laboratory Medicine, UCSF, San Francisco, California, USA.; 5 University of British Columbia, Victoria, British Columbia, Canada.; 6 Bloodworks Northwest, Seattle, Washington, USA.; 7 New York Blood Center Enterprises, New York, New York, USA.; 8 Vitalant, Scottsdale, Arizona, USA.; 9 Department of Medicine, UCSF, San Francisco, California, USA.; 10 See Supplemental Acknowledgments for REDS-IV-P details.

**Keywords:** COVID-19, Clinical practice, Molecular diagnosis

## Abstract

Respiratory viruses such as influenza do not typically cause viremia; however, SARS-CoV-2 has been detected in the blood of COVID-19 patients with mild and severe symptoms. Detection of SARS-CoV-2 in blood raises questions about its role in pathogenesis as well as transfusion safety concerns. Blood donor reports of symptoms or a diagnosis of COVID-19 after donation (post-donation information, PDI) preceded or coincided with increased general population COVID-19 mortality. Plasma samples from 2,250 blood donors who reported possible COVID-19–related PDI were tested for the presence of SARS-CoV-2 RNA. Detection of RNAemia peaked at 9%–15% of PDI donors in late 2020 to early 2021 and fell to approximately 4% after implementation of widespread vaccination in the population. RNAemic donors were 1.2- to 1.4-fold more likely to report cough or shortness of breath and 1.8-fold more likely to report change in taste or smell compared with infected donors without detectable RNAemia. No infectious virus was detected in plasma from RNAemic donors; inoculation of permissive cell lines produced less than 0.7–7 plaque-forming units (PFU)/mL and in susceptible mice less than 100 PFU/mL in RNA-positive plasma based on limits of detection in these models. These findings suggest that blood transfusions are highly unlikely to transmit SARS-CoV-2 infection.

## Graphical abstract

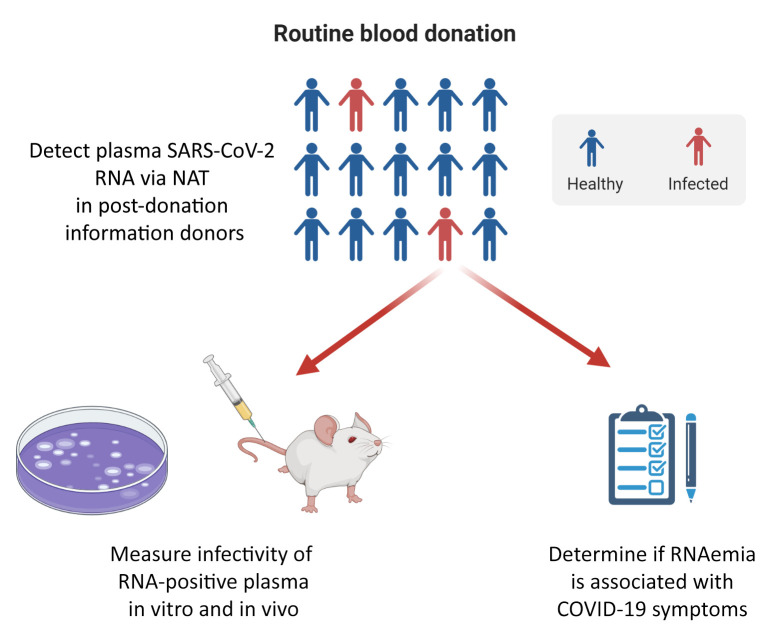

## Introduction

Although routine blood donation screening has rendered the blood supply extraordinarily safe with regard to tested infectious agents such as HIV and hepatitis B and C, in recent decades multiple infectious disease outbreaks have posed potential risks to the blood supply (1). These agents often originate as zoonoses and have a demonstrated capacity to spread rapidly between animal and human populations, and include respiratory viruses such as H1N1 influenza, SARS, MERS, and most recently SARS-CoV-2 (2). The attributes for an infectious agent to be transfusion transmissible include its presence in blood during an asymptomatic phase in the donor, survival in blood during processing and storage, infection in the recipient, and to be identified as clinically relevant, causing apparent disease. To ensure an adequate blood supply during a pandemic, blood establishments must consider whether blood collected during an outbreak may contain the infectious agent given that transfusion recipients are more susceptible to severe disease and serious sequelae.

In addition to screening, donor selection and questioning are in place to remove donors with recognized risk factors and potentially reduce the threat of infectious agents for which no assay is available or testing routinely performed. Many blood centers have procedures for collection of post-donation information (PDI) and retrieval of nontransfused blood products from donors who develop clinical symptoms, are diagnosed with an infection, or who recall risk information shortly after donation. PDI likely prevents transfusion-transmissible infections (TTIs) by allowing for retrieval of potentially contaminated units. PDI reports and testing of plasma units manufactured from these donations allows tracking of seasonal patterns of respiratory infections, including fever and general influenza-like illness (3). In 2020, PDI reporting expanded to include COVID-19–related symptoms or confirmed infection and is being used to monitor seasonal changes in PDI reporting rates relative to SARS-CoV-2 outbreaks. While the risk of transfusion-transmitted SARS-CoV-2 is thought to be low, several studies have noted detectable viral RNA in blood. SARS-CoV-2 RNAemia in hospitalized patients with critical disease ranges from 15% to 90%, compared with that in outpatients with mild disease ranging from 2% to 13% (4). In presymptomatic blood donors who were diagnosed with or developed symptoms of COVID-19 within 15 days of donation, 1% prevalence of SARS-CoV-2 RNAemia has been reported (5). A few studies have attempted to determine whether RNA-positive blood samples harbor infectivity using cellular models, all with negative results (4–8). This suggests that for SARS-CoV-2 RNAemia may not indicate the presence of infectious virions, consistent with what is known of other coronaviruses (9). The sensitivity of these models, however, is limited (10), making it difficult to rule out risk of transfusion transmission, particularly in more vulnerable transfusion recipients.

In this study, we investigated SARS-CoV-2 RNA and antibody reactivity in plasma from over 2,000 donors reporting SARS-CoV-2–compatible symptoms or diagnosis within 2 weeks after donation through routine blood center operational procedures at blood collection organizations collecting approximately 50% of the US blood supply. Using highly sensitive cellular and animal models, we evaluated risk of infection via intravenous (i.v.) exposure and screened RNA-positive plasma with the highest viral load for infectious virions.

## Results

### Rate of PDI during pre– and post–COVID-19 periods.

**Figure 1:**
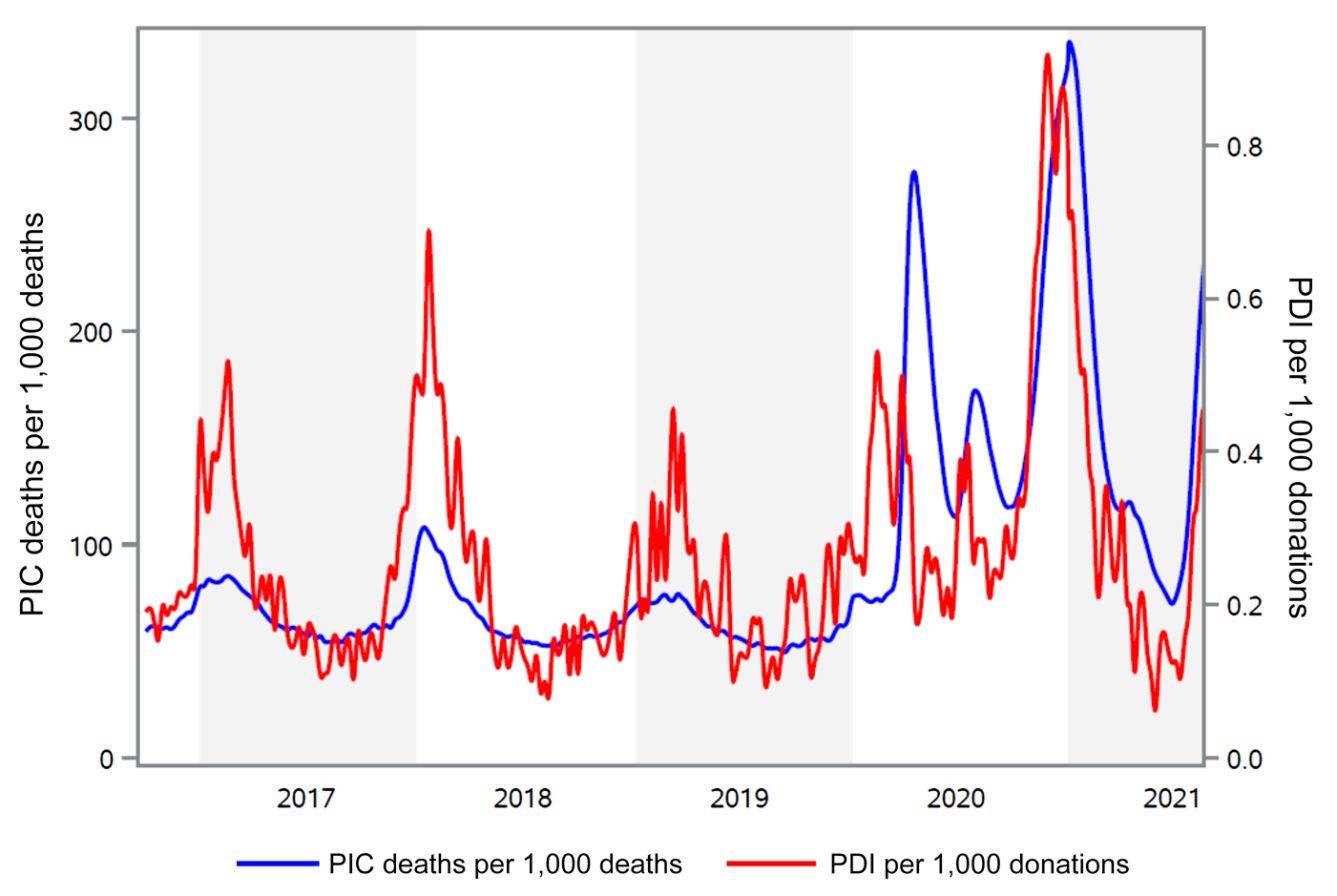
Comparison of the PDI rate with reported mortality due to pneumonia, influenza, and COVID-19. The red line represents the rate of PDI reports per 1,000 donations to the American Red Cross from week 40, 2016 through week 31, 2021 (right axis). The blue line shows data published by the CDC for the number of deaths from pneumonia, influenza, or COVID-19 per 1,000 deaths (PIC, left axis). CDC data were obtained from the website https://www.cdc.gov/flu/weekly/index.htm

PDI reports from the fourth quarter of 2016 through the end of July 2021 from the American Red Cross (representing approximately 40% of the US blood supply) were plotted and cross-referenced with public health data on pneumonia-, influenza-, and COVID-19–related mortality for the same period. From 2016 to 2019, the weekly rate of PDI reports ranged from 0.06 to 0.94 per 1,000 donations. A marked seasonality of PDI cases was evident; peaks coincided with mortality surveillance relating to pneumonia and influenza in winter months (Figure 1). In 2020 there were 3 additional peaks in PDI reports occurring in March, July, and November, the last doubling the 2 previous peaks in magnitude and duration. The ramp up of the fourth wave of COVID-19 associated with the Delta variant is also evident in the second quarter of 2021. These data show that PDI data mirror public health reporting of influenza and pneumonia mortality, and spikes in PDI reports coincided with or preceded the observed increase in COVID-19–related mortality during the most recent SARS-CoV-2 pandemic waves.

### Detection of SARS-CoV-2 RNA and antibodies in quarantined plasma units from donors reporting PDI.

**Figure 2:**
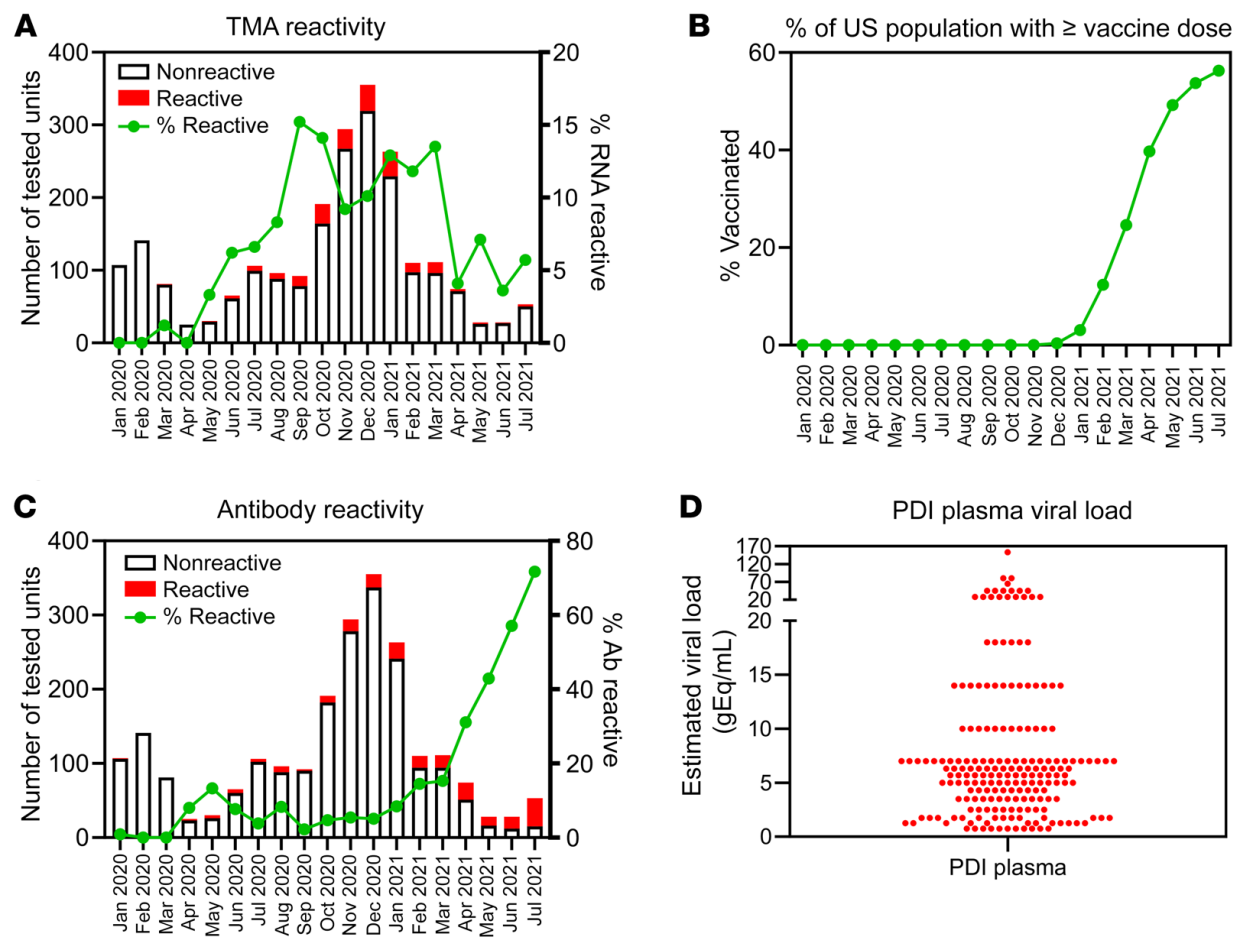
Detection of SARS-CoV-2 RNA and antibodies in PDI donors. (**A**) Plasma samples (*n =* 2,250) were collected over the course of 19 months throughout the United States. Bars correspond to total number of donations tested for SARS-CoV-2 RNA per month of collection, with the red portion denoting those reactive. Line corresponds to percentage of RNA-reactive donations. (**B**) Data published on the CDC website (https://data.cdc.gov/Vaccinations/COVID-19-Vaccination-Trends-in-the-United-States-N/rh2h-3yt2) show the average proportion of the US population that had received at least 1 vaccine dose for each month of the period during which PDI plasmas were tested. (**C**) Plasma samples (*n =* 2,250) were collected over the course of 19 months throughout the United States. Bars correspond to total number of donations tested for SARS-CoV-2 spike antibodies per month of collection, with the red portion denoting those reactive. The green line corresponds to percentage of antibody-reactive donations. (**D**) Estimated viral load of SARS-CoV-2 RNA in 196 reactive plasma samples based on TMA reactivity in replicates normalized to a standard curve.

This study identified 2,250 donors reporting COVID-19–related PDI cases with available quarantined frozen plasma units collected from January 2020 through July 2021 throughout the United States across 4 blood collection organizations: the American Red Cross, Vitalant, the New York Blood Center, and Bloodworks Northwest. Samples from these quarantined plasma units were tested in singlet for SARS-CoV-2 RNA and antibodies. Initially RNA-reactive samples were retested in 11 replicates and considered RNA repeat reactive based on transcription-mediated amplification (TMA) reactivity in at least 1 of those 11 replicates. Of the 2,250 samples tested, 196 (8.7% [95% CI 7.5–9.9]) were RNA repeat reactive. In March 2020, RNA prevalence in plasma from PDI donors was 1%, gradually increasing to 3%–8% during May–August 2020 and to 9%–15% from September 2020 to March 2021. From April to July 2021, plasma RNA prevalence sharply fell to approximately 4% (Figure 2A), coincident with widespread introduction of vaccination in the community (Figure 2B) and a sharp increase in antibody prevalence in the PDI donors (Figure 2C). Viral loads in RNA-reactive plasma units were estimated based on the number of reactive tests among 12 replicate tests using a standard curve generated with heat-inactivated virus (Supplemental Figure 1; supplemental material available online with this article; https://doi.org/10.1172/JCI159876DS1). Median estimated viral load in the 196 RNA repeat-reactive plasma samples was 6 gEq/mL, with 90% of samples having an estimated viral load of 18 gEq/mL or lower (Figure 2D). Samples were also tested after 16-fold dilution to mimic testing of pools of samples, a testing strategy commonly used for nucleic acid blood donation screening for transfusion-transmitted agents. Of 190 repeat-reactive samples available for dilution testing, only 18 were reactive in a single test after 16-fold dilution, implying that testing 16-sample pools would have missed 90% of the repeat-reactive samples due to low plasma viral load. All but 2 of the 196 RNA repeat-reactive samples were negative when tested for SARS-CoV-2 spike antibody. RNA-positive and -negative PDI units were distributed widely across the United States in the regions covered by the participating blood centers (Supplemental Figure 2).

**Figure 3:**
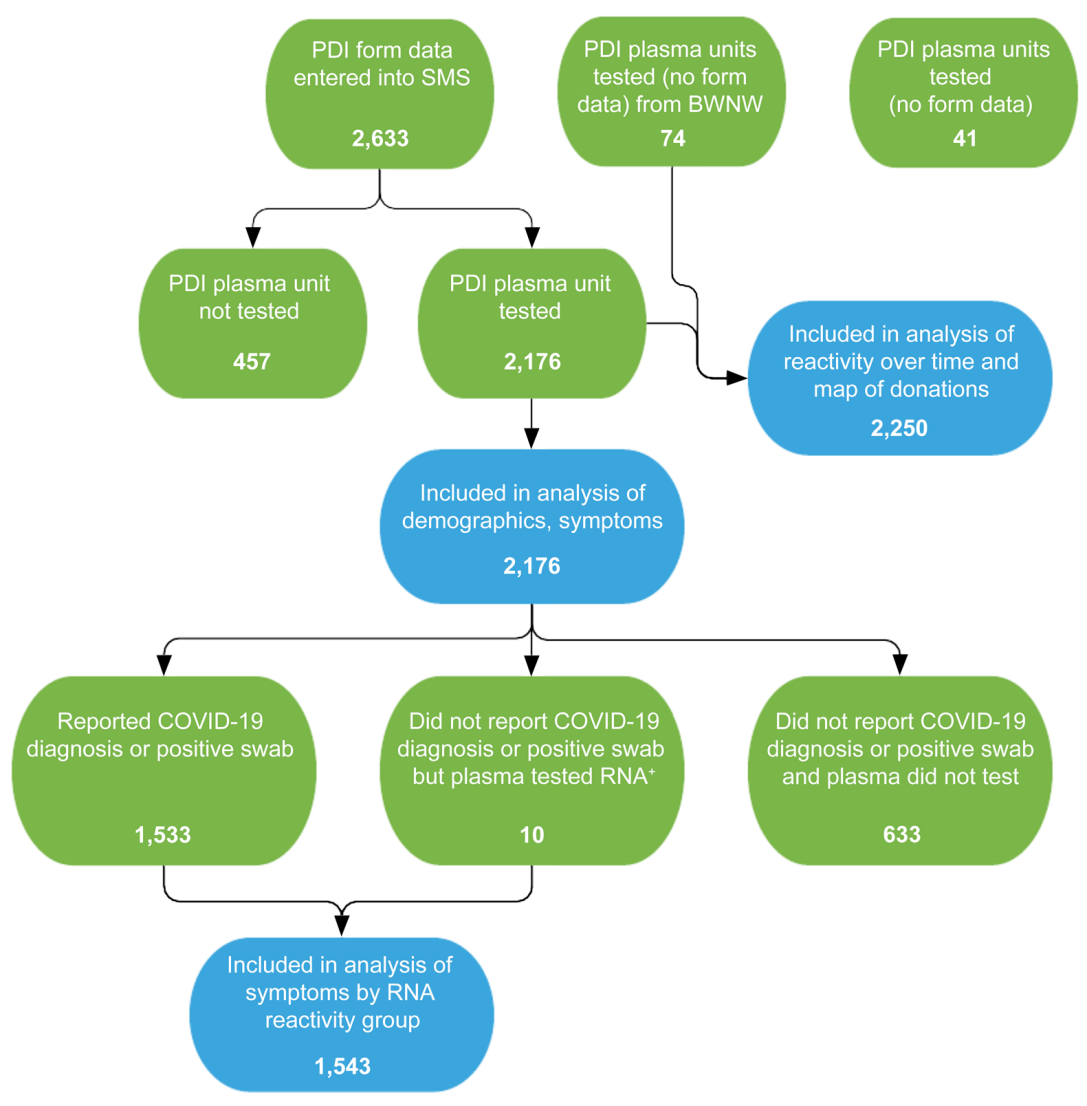
Flowchart of PDI donor enrollment and analytic groups. PDI donor questionnaires were entered into the study management system (SMS). Questionnaires were not obtained from PDI donors at Bloodworks Northwest (BWNW). A total of 2,250 plasma units were available for SARS-CoV-2 RNA testing, and 2,176 had questionnaire data for analysis of demographics and symptoms. Of these donors, 1,543 were determined to be SARS-CoV-2 infected based on a self-report of a positive SARS-CoV-2 clinical test or by detection of RNAemia in the donor’s plasma unit. Green bubbles indicate the parent populations and blue bubbles indicate analysis populations.

While the TMA assay is highly specific, it is possible that some of the repeat-reactive plasma samples tested falsely positive. During the early period of the COVID-19 pandemic, in an effort to identify potential COVID-19 convalescent plasma donors and encourage blood donation during a severe supply shortage, blood donors at participating blood collection organizations were universally screened for anti-spike antibodies, from approximately June 2020 through June 2021. We were able to follow the trajectory of antibody evolution via universal serological screening of blood donors in 132 of 196 donors with detectable SARS-CoV-2 RNA (11). These results showed that all RNAemic PDI donors with available longitudinal antibody test data evolved antibody responses to spike and nucleocapsid proteins (Supplemental Figure 3). The donors most likely to have a false positive plasma TMA test were the 10 who tested TMA positive without a known diagnosis of COVID-19 (Figure 3). Longitudinal antibody testing was available for 8 of these 10 donors, and all 8 seroconverted. Additionally, the mean number of positive replicate tests for these 10 donors was 9.7 (range 4–12), implying that these donors all had true positive testing for RNAemia. Finally, we previously published an analysis of 17,995 minipools of 6 to 16 plasma samples from nonselected blood donors who were tested using the same TMA assay. Of 11 initially reactive minipools, 8 were found not to be repeat reactive using a supplemental TMA assay, yielding a potential false positive rate of 4.4 per 10,000 tests (6).

### Relationship between symptomatic infection and plasma SARS-CoV-2 RNA detection.

With the onset of the COVID-19 pandemic, 3 of the blood collection organizations in this study implemented enhanced PDI questionnaires to include 12 COVID-19–related symptoms (Table 1). A total of 2,176 donors reported a positive test for SARS-CoV-2 or COVID-19 symptoms within 2 weeks of donation (Supplemental Table 1). The majority of donors reporting PDI were White, non-Hispanic, female, and between 30 and 64 years of age (Table 1); PDI donor demographics mirrored those of the underlying blood donor population. Of the 2,176 PDI donors with questionnaire responses, 1,533 (70%) reported being diagnosed with COVID-19 or a positive nucleic acid or antigen test, 10 (0.6%) were identified by RNA screening of a sample of their plasma unit in the absence of reporting a positive COVID-19 swab test or diagnosis, and 633 (29%) reported 2 or more symptoms consistent with COVID-19 in the absence of a positive diagnosis or test result. Donors in the last group were interpreted as either having undiagnosed COVID-19 or having symptomatic illness due to an indeterminate cause (Figure 3). Of 1,533 donors who reported a positive diagnostic test result, 184 (12.0%) were TMA reactive versus 10 of 643 (1.6%) of donors who reported symptoms only, making the chances of finding RNAemia in donors with a positive diagnostic test 7.5 times greater (*P <* 0.0001, Fisher’s exact test) than in symptomatic donors without a positive diagnostic test result.

**Figure 4:**
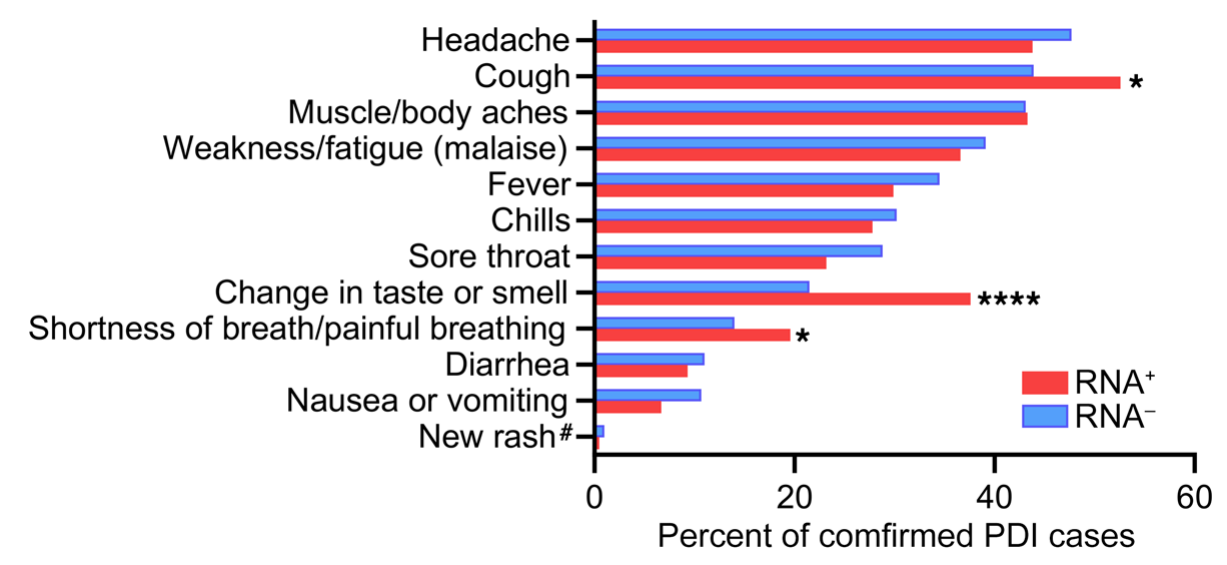
Symptom distribution in RNAemic and non-RNAemic PDI donors. A questionnaire detailing the presence of 12 symptoms in the 7 days after blood donation was administered. Data from PDI donors whose plasma tested SARS-CoV-2 RNA–positive (*n =* 194) and RNA–negative (*N =* 1,349) are shown. Symptom frequency was compared using a 2-tailed χ^2^ test; *P < 0.05, ****P < 0.0001. ^#^Excluding rash at the phlebotomy site.

Of the 1,533 PDI donors who reported a positive diagnosis or test result, 468 (30%) were asymptomatic or reported only 1 symptom, consistent with COVID-19 within 7 days of their donation. The most frequent symptoms among the 1,543 donors with confirmed COVID-19 infection were headache, cough, muscle or body ache, and weakness or fatigue, which were reported in 39% to 47% of donors (Figure 4). Because RNAemia has been associated with more severe disease in some reports (12), we tested whether symptoms varied by RNA detection status, acknowledging that disease severity is not equivalent to number or type of symptoms. The mean number of symptoms in individual PDI donors was 3.3 (SD 2.5) and did not differ based on RNAemia status. Compared with non-RNAemic donors, RNAemic donors were significantly more likely to report cough, shortness of breath or painful breathing, and loss of taste or smell (Table 2). No correlation was found between ABO blood type and RNAemia or symptom severity.

### Infectivity of SARS-CoV-2 RNA–positive PDI plasma.

**Figure 5:**
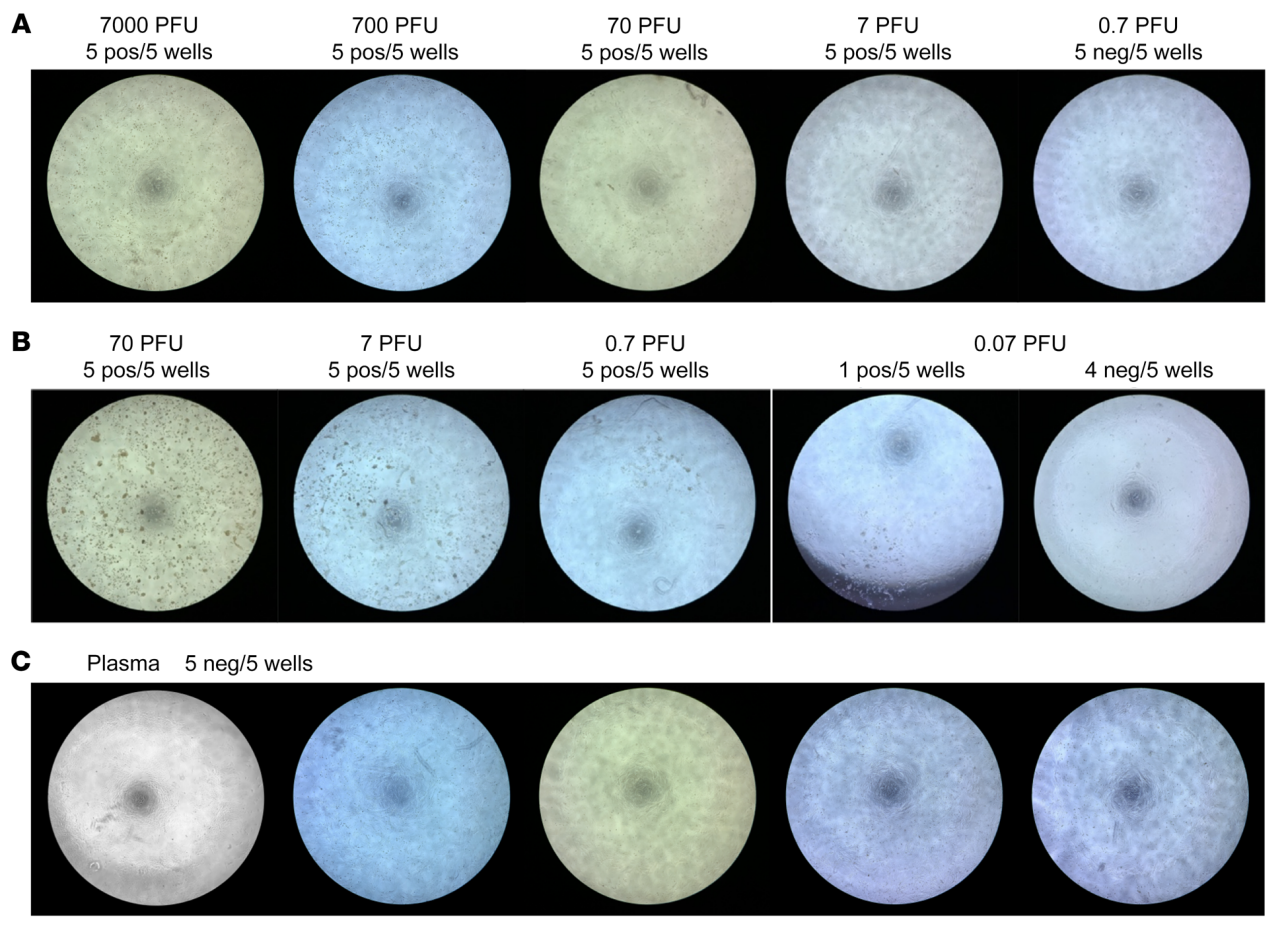
No viral replication after culture of SARS-CoV2 RNA–positive human plasma in susceptible cell lines. (**A**) Vero-TMPRSS2 cells in 96-well plates were infected with indicated doses of SARS-CoV-2. Five replicate wells were tested for each dosage. Two days after infection, weak CPE developed in all wells infected with as low as 7 PFU/well, and no CPE developed in all wells infected with 0.7 PFU/well (representative wells shown). (**B**) Vero-ACE2-TMPRSS2 cells were tested as in **A**. Two days after infection, clear CPE developed in all wells infected with as low as 0.7 PFU and 1 out of 5 wells infected with 0.07 PFU developed CPE. (**C**) Vero-ACE2-TMPRSS2 cells were incubated with 5 different SARS-CoV-2 RNA–positive human plasmas in 5 replicate wells each for 2 hours at 37°C before washing and incubation with fresh medium. No CPE developed in any wells at 3 days after infection.

Although the viral load detected in RNA-positive plasma units was low, it is still possible that SARS-CoV-2 in these units could be transfusion transmissible. To address this theoretical risk we tested the ability of plasma from RNA-positive units to infect a permissive cell line in vitro and an engineered mouse model in vivo. Previously published work tested the infectivity of plasma from RNAemic blood donors using Vero E6 cells (4, 7) or Vero E6 cells expressing the spike priming protein TMPRSS2 (6); none of the tested units were found to be able to infect the cell lines. We tested the sensitivity of a Vero cell line expressing TMPRSS2 (Vero-TMPRSS2 cells) by measuring cytopathic effect (CPE) 2 days after inoculation with the WA1 strain of SARS-CoV-2, and CPE was detectable after inoculation with 7 plaque-forming units (PFU) but not with 0.7 PFU of virus (Figure 5A). We next modified Vero-TMPRSS2 cells to coexpress the SARS-CoV-2 receptor ACE2 (Vero-ACE2-TMPRSS2 cells). The Vero-ACE2-TMPRSS2 cell line was at least 10-fold more susceptible than the Vero-TMPRSS2 cells, with CPE yielding larger clumps of cells, and CPE detected in 1 of 5 wells after inoculation of 0.07 PFU of virus (Figure 5B). The difference in susceptibility to SARS-CoV-2 infection between Vero-ACE2-TMPRSS2 and Vero-TMPRSS2 persisted up to 7 days after infection (data not shown). Six PDI plasma unit samples from those with the highest viral loads (45–150 gEq/mL) were selected for inoculation of the Vero-ACE2-TMPRSS2 cells undiluted and serially diluted in DMEM supplemented with 2% FBS for 2 hours before the cells were washed and cultured in fresh medium. The unit with the highest RNA level caused cell death, and the remaining 5 units were tested in culture. None of the wells showed CPE 3 days after inoculation (Figure 5C). Cell culture supernatants were collected for total RNA extraction followed by qRT-PCR for quantification of SARS-CoV-2 RNA. No SARS-CoV-2 RNA was detected from the culture supernatants of Vero-ACE2-TMPRSS2 inoculated with RNA-positive plasma samples. Based on the in vitro infection data, we conclude that the RNA-positive plasma units tested contained less than 0.7 to 7 PFU/mL of infectious SARS-CoV-2.

To further probe the transfusion-transmission risk of RNAemic donors, a susceptible mouse model was established to test the same plasma units. In addition, to evaluate the risk associated with i.v. exposure to a known infectious product, lab-cultured SARS-CoV-2 variant B.1.1.7 (Alpha variant) was chosen as this strain was dominant in the United States at the time the RNA-positive donor samples were collected. Initial infection experiments using a knockin mouse model (in which the human *ACE2* gene is under the endogenous mouse *ACE2* promoter on a B6 background) in combination with pretreatment with an anti-IFNAR antibody to increase susceptibility, showed detectable virus in the oropharynx but no clinical effect after intranasal (i.n.) or i.v. inoculation of lab-cultivated B1.1.7 virus at doses up to 1.1 × 10^4^ PFU (data not shown). We next evaluated the K18-hACE2–transgenic (K18-hACE2–Tg) model (in which the human *ACE2* gene is under the control of the human keratin 18 promoter) in the IFNAR-knockout (IFNAR-KO) B6 background for susceptibility to infection. These mice displayed high susceptibility to i.n. infection with lab-cultivated B1.1.7 virus at a wide range of doses, resulting in severe weight loss (Supplemental Figure 4A), labored breathing and lethargy, and ultimately, death (Supplemental Figure 4B). These mice also had high levels of viral RNA detected from oropharyngeal swabs at all time points after infection, except for a single sample collected after death (Supplemental Figure 4C). Viral RNA was also detected on day 2 in the blood of 5 of 6 of these mice (Supplemental Figure 4D).

**Figure 6:**
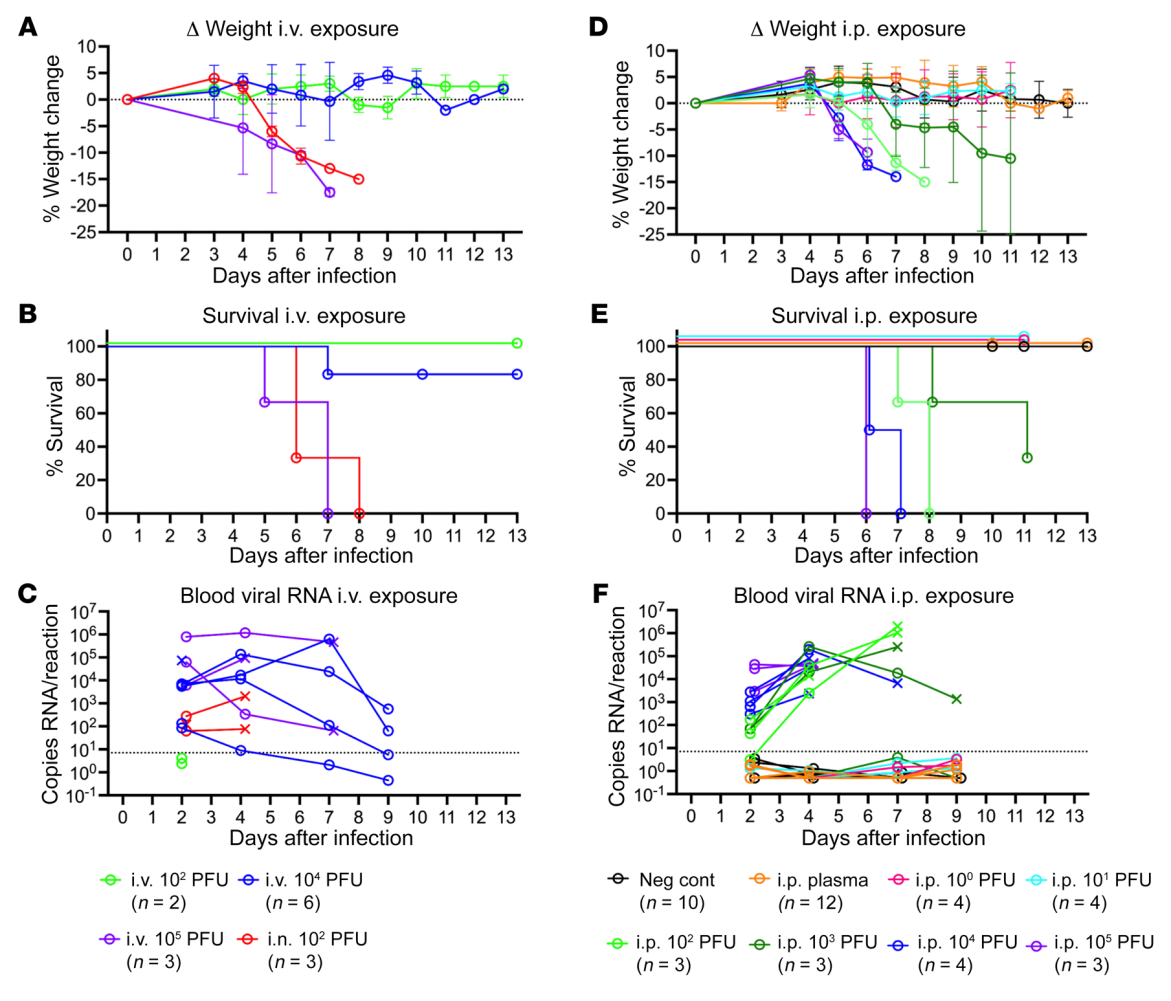
No infection observed with exposure to SARS-CoV2 RNA–positive human plasma. (**A**–**C**) K18-hACE2–Tg IFNAR-KO mice were infected with 1.1 × 10^2^ PFU, 1.1 × 10^4^ PFU, or 1.1 × 10^5^ PFU intravenously, or 1.1 × 10^2^ PFU intranasally with the B1.1.7 variant of SARS-CoV-2. (**D**–**F**) K18-hACE2–Tg IFNAR-KO mice were infected with 1.1 × 10^0^ PFU, 1.1 × 10^1^ PFU, 1.1 × 10^2^ PFU, 1.1 × 10^3^ PFU, 1.1 × 10^4^ PFU, or 1.1 × 10^5^ PFU intraperitoneally with the B1.1.7 variant of SARS-CoV-2 or given 500 μL SARS-CoV2 RNA–positive human plasma intraperitoneally (6 samples into 2 mice each were tested). Unexposed IFNAR-KO littermates that do not carry the K18-hACE gene (noncarriers) were used as negative controls. (**A** and **D**) Weights were measured daily and the percentage weight change was calculated for each mouse over time, with mean change in weights and standard deviation plotted for each group. (**B** and **E**) Percentage survival over time by group. (**C** and **F**) At indicated time points, 20 μL of EDTA whole blood was collected, RNA was isolated, and SARS-CoV2 RNA levels were measured by qRT-PCR. Values are plotted for each mouse. × indicates nonsurviving mouse. Dashed line indicates maximum value detected among 63 negative blood sample controls plus 0.5 and was used as a cutoff for positive signal. When no viral RNA was detected, a value of 0.5 was assigned.

With a highly susceptible mouse model established, we next evaluated the infectivity of the i.v. route of exposure. K18-hACE2–Tg IFNAR-KO mice were inoculated with lab-cultivated SARS-CoV-2 B1.1.7 at a dose of 1.1 × 10^2^ PFU, 1.1 × 10^4^ PFU, or 1.1 × 10^5^ PFU i.v., or 1.1 × 10^2^ PFU i.n. as a positive control. All 3 mice at 1.1 × 10^5^ PFU, 1 of 6 mice at 1.1 × 10^4^ PFU, and 3 of 3 positive control mice had weight loss (Figure 6A), labored breathing and lethargy, and death (Figure 6B). Neither of the 2 mice experienced any symptoms at the 1.1 × 10^2^ PFU i.v. dose. High blood viral levels were seen with the 1.1 × 10^5^ PFU and 1.1 × 10^4^ PFU doses, even in the absence of other symptoms (Figure 6C). Oropharyngeal swabs were not included in all experiments, as viral RNA was not detected in these samples after i.v. or intraperitoneal (i.p.) exposure in our initial experiment except for 1 i.v. exposure mouse sample collected after death (Supplemental Figure 5). Attempts to administer high doses (0.5 mL) of plasma from RNAemic donors i.v. led to rapid death in most cases, and the same result was seen using 0.5 mL RNA-negative human plasma or 0.5 mL 40% citrate-phosphate-dextrose-adenine 1 (CPDA-1) in PBS (data not shown). This suggests that the volume of anticoagulant given through rapid i.v. injection was toxic, as 0.5 mL i.v. infusions are generally well tolerated by mice. To address this limitation the plasma was administered i.p. to allow for slower absorption into the bloodstream, and several doses of lab-cultivated SARS-CoV-2 B1.1.7 administered i.p. were included as controls.

K18-hACE2–Tg IFNAR-KO mice were given 0.5 mL of RNA-positive plasma i.p. (6 different units given to 2 mice each for a total *n =* 12), and controls were given lab-cultivated SARS-CoV-2 B1.1.7 i.p. at a dose of 1.1 PFU (*n =* 4), 11 PFU (*n =* 4), 1.1 × 10^2^ PFU (*n =* 3), 1.1 × 10^3^ PFU (*n =* 3), 1.1 × 10^4^ PFU (*n =* 4), or 1.1 × 10^5^ PFU (*n =* 3). An additional negative control group of unexposed non–hACE2-carrier littermates were included (*n =* 10). Mice that received the RNA-positive plasma displayed no signs of infection, including no weight loss (Figure 6D), death (Figure 6E), or circulating viral RNA (Figure 6F). In contrast, 3 of 3 of the 1.1 × 10^2^ PFU, 2 of 3 of the 1.1 × 10^3^ PFU, 4 of 4 of the 1.1 × 10^4^ PFU, and 3 of 3 of the 1.1 × 10^5^ PFU dose i.p. groups experienced weight loss, persistent viremia, and death. No signs of disease were observed at 1.1 or 11 PFU given i.p. Based on the in vivo infection data, we would conclude that the RNA-positive plasma units tested contained less than 100 PFU/mL of infectious SARS-CoV-2.

A potential concern with the plasma transfusion experiments in comparison with the lab-cultivated virus inoculation is that the plasma itself may be inactivating the virus. Furthermore, the RNA-positive plasma samples underwent a total of 3 freeze-thaw cycles between collection and administration to the mice, which also could have affected infectivity. To address these concerns, 2 aliquots of one of the RNA-positive plasma samples used above were utilized along with 2 aliquots of the lab-cultivated B1.1.7 virus lot used in the above experiments. One of each was thawed, used to prepare serial dilutions in plasma, and subjected to 2 additional freeze-thaw cycles for a total of 3. The others were thawed on the day of infection and used to prepare fresh serial dilutions in plasma or PBS. K18-hACE2–Tg IFNAR-KO mice were given 0.5 mL of virus diluted in SARS-CoV-2 RNA-positive plasma i.p. at a dose of 1.1 × 10^2^ PFU, 1.1 × 10^3^ PFU, or 1.1 × 10^4^ PFU (either prepared fresh or after 2 extra freeze-thaw cycles). An additional group received 1.1 × 10^2^ PFU prepared fresh in PBS (as done previously), and a negative control group of unexposed non–hACE2-carrier littermates were included (Supplemental Figure 6A). While clear differences were again observed by dose, no meaningful differences were observed with additional freeze-thaw cycles or between plasma and PBS vehicle (Supplemental Figure 6, B–D).

## Discussion

In this study, we found that the rate of PDI reporting follows a seasonal pattern mirroring that of public health reports of respiratory infections. This overlapping pattern has been maintained during successive waves of the COVID-19 pandemic. Testing of plasma samples from index donations from over 2,000 PDI donors revealed that RNA was detected almost exclusively in donors who lacked anti-spike antibodies, and the rate of RNAemia decreased after widespread COVID-19 vaccine administration. Validation of the mouse model of infection revealed that mice were much more susceptible to a given dose of i.n. SARS-CoV-2 infection compared with the i.v. route, theoretically decreasing the potential risk of blood-borne transmission. Examination of the PDI plasma units with the highest levels of detected viral RNA revealed no evidence of infectious virus after inoculation of sensitive target cell lines or in a permissive mouse model.

Detection of viral RNA in the blood after a respiratory infection is uncommon across most viruses. A study of over 1,000 historical PDI donors with influenza-like illness revealed no viral RNA in blood collected (3). More recently, H3N2 influenza viral RNA was detected in 1 of 28 plasma samples from presymptomatic donors who reported PDI up to 14 days after donation (13). Since no experiments were performed to determine infectivity in the RNA-positive plasma, and in the absence of documented cases of transfusion-transmitted influenza, the risk of transfusion transmission of influenza virus remains theoretical. Novel coronaviruses appear to behave differently from influenza. Low levels of MERS-CoV RNA have been detected in approximately 50% of serum samples tested within 1 week of diagnosis, and paired viral isolation attempts were unsuccessful (14). SARS-CoV-1 RNA was detected during the first week of illness in plasma of 78% to 79% adults and 87.5% children infected with SARS-CoV-1, albeit in very low copy numbers (15, 16). The presence of RNAemia in COVID-19 has been associated with disease severity and peripheral markers of inflammation, and higher level viremia is associated with mortality (12, 17–21). Our data show that among COVID-19 patients with mild disease, RNAemia is more frequently associated with respiratory symptoms and loss of taste and/or smell, potentially implying a different mechanism of pathogenesis if the virus is systemically detectable in contrast to an infection localized to mucosal surfaces. It is known that naive individuals are much more susceptible to severe disease than vaccinated individuals (22, 23). The fact that RNAemia was only very rarely detected in participants with preexisting antibody responses to SARS-CoV-2 suggests that one mechanism by which naive individuals are more susceptible to severe COVID-19 may be due to inability to prevent systemic invasion of the virus. Prior studies have found that blood group O participants were less commonly infected with SARS-CoV-2 (24, 25). In the current study, there was no association observed between blood ABO group and RNAemia or symptoms, implying that the influence ABO group may have on disease acquisition did not extend to disease severity after infection in our population.

Prevalence of SARS-CoV-2 RNA in plasma from presymptomatic or asymptomatic blood donors is higher than previously described in unselected, non-PDI blood donors (6, 8) and comparable to the estimate in patients with mild disease. Estimated viral loads in plasma in our study were low, generally under 20 gEq/mL. The low levels of viral load detected in PDI donors make detection of RNA challenging using pooled samples; our data suggest that pooling of samples prior to testing for SARS-CoV-2 could result in an approximately 90% underestimate in RNA detection given the low level of SARS-CoV-2 RNA in plasma. Testing of pooled samples revealed very low rates of RNAemia in blood donors (6, 8), and no transfusion-transmitted cases have been reported to date (26). Lack of antibody reactivity in RNAemic donors reporting PDI consistent with COVID-19 suggests these samples were obtained at an early stage of acute infection. Given the decreased rate of RNAemia detected after widespread introduction of vaccination and increasing antibody prevalence in blood donors, it is tempting to speculate that prior vaccination may prevent RNAemia, even in breakthrough infections. Ongoing follow-up studies are required to answer this question.

Several in vitro and in vivo models are available to study SARS-CoV-2; these include cell lines, organoids, and animal models (reviewed in ref. 27). Cell lines expressing the SARS-CoV-2 receptor ACE2 are permissive to SARS-CoV-2 and sustain viral replication; however, they present some caveats that should be considered when selecting the appropriate line for the question under investigation. Monkey kidney Vero E6 cells yield high viral titers, thus enabling rapid drug screening studies, but they may behave differently than human airway cells (28). In this study, in vitro infectivity of SARS-CoV-2 RNA from human plasma was assayed using Vero-ACE2-TMPRSS2 cells, which were orders of magnitude more susceptible compared with Vero-ACE2 cells. In spite of the high sensitivity of this cell line to infection with SARS-CoV-2, and our selection of the highest-titer RNA-positive plasma samples from our cohort, we did not detect any infection following exposure to RNA-positive plasma.

Different animal species have been shown to be susceptible to SARS-CoV-2, from small rodents to nonhuman primates. While wild-type strains of mice are not very susceptible to most circulating strains of SARS-CoV-2, adding human ACE2 to mice either through transient induction (29, 30), knockin models (31), or transgenic systems have all been shown to increase susceptibility. The K18-hACE2–Tg mouse, first developed for studies of SARS-CoV-1 (32), has been used most extensively, leading to symptomatic and sometimes lethal disease dependent on viral stain and dose used (33, 34). In our studies, we utilized the K18-hACE2–Tg mice on an IFNAR-KO background to maximize susceptibility to infection. Using this highly sensitive model system, we evaluated the relative risk of infection following exposure to SARS-CoV-2 through i.v., i.n., and i.p. routes and found that i.v. exposure had the lowest risk. Infection through i.v. exposure required 10^4^–10^5^ PFU, which is at least 2 to 3 logs more than was needed to infect through the other routes, and well beyond the doses detected in our donors, which were generally less than 100 gEq/mL or approximately 0.1 PFU/mL. Using the more susceptible i.p. route, we saw no signs of viral replication or illness in mice following exposure to the highest-titer RNA-positive plasma samples. Failure to infect is likely due to the low level of virus in plasma (<1 PFU, with 100 PFU being the lowest dose at which we detected infection after i.p. exposure to cultured virus). The combination of the lack of infectivity in vitro and in vivo of the RNA-positive plasma, the low levels of RNA detected in these samples, and the poor transmission of infectious SARS-CoV-2 via i.v. routes all suggest little to no risk of transfusion transmission of this virus.

The current study has some limitations. Only plasma samples were tested, so if infectious virus were enriched in cellular blood components that would not have been detected. Symptom responses were self-reported. Not all questions had responses; the lowest rate was for “new rash,” which only had a 63% response rate. A reasonable interpretation is that no response indicated absence of a symptom, especially for COVID-19–specific symptoms with extensive media coverage. Another limitation is that the PDI questionnaire was modified during the pandemic to include additional questions as new COVID-19–related symptoms were described. In addition, tracing blood products from PDI donors that were transfused prior to being interdicted and quarantined to examine whether or not transfusion recipients were infected with SARS-CoV-2 was not performed, as this is not standard blood center practice. Study of a product drawn from a donor who developed COVID-19 shortly after donation revealed no infection of an immunocompromised recipient of a platelet unit from the PDI donor, although it is difficult to draw conclusions from a single case report (35). A limitation of the K18-hACE2–Tg mouse model is that the expression of the transgene is regulated by the human keratin 18 promoter, which directs expression to epithelia. This includes epithelia of the airway and many other tissues, enabling efficient infection, but as it is not the normal expression profile for ACE2, it is possible that it could lead to altered susceptibility to i.v. exposure. Additionally, due to the toxicity of the anticoagulant, plasma units were inoculated i.p. rather than i.v.

In summary, this study, which was conducted at blood collection organizations representing approximately 50% of the US blood supply, with over 2,000 blood donors reporting COVID-19 symptoms or infection shortly after donation, revealed a number of important findings. First, spikes in blood donor PDI reports preceded or mirrored COVID-19–associated mortality trends at the national level, demonstrating the value of the blood donor population for infection surveillance. Second, RNAemia was detectable in approximately 8% of PDI donation samples but was seen only rarely in individuals who possessed anti–SARS-CoV-2 antibodies. Third, in this cohort of participants with relatively mild disease, several COVID-19 symptoms, including respiratory and the highly specific anosmia/ageusia, were more common in those with RNAemia. Finally, using well-validated cell culture and mouse models, none of the plasma samples with detectable SARS-CoV-2 RNA were able to induce infection. These findings suggest that blood transfusions are highly unlikely to transmit SARS-CoV-2 infection and support the current policy of not testing blood donor products for SARS-CoV-2 RNA.

## Methods

### Study participants and data collection.

Blood collection organizations collect PDI as part of their standard procedures. During the COVID-19 pandemic, participating blood collection organizations collected additional standardized information (enhanced PDI) on donors who reported PDI and met the study eligibility criteria. The additional information was collected through a web-based questionnaire used to assess whether the PDI donors’ symptoms were consistent with SARS-CoV-2 infection. Staff completed the enhanced PDI questionnaire during a phone call with the donor or by abstraction/extraction from blood collection organizations’ operational PDI database. Plasma components from donations with relevant PDI were quarantined and sent to the testing laboratory for TMA and serological testing. The study population included blood donors who self-reported a COVID-19 diagnosis or positive test result within 14 days of donation and those who reported at least 2 potential COVID-19 symptoms within 7 days of donation. These symptoms were limited to fever, cough, shortness of breath or difficulty breathing, chills, muscle pain, headache, sore throat, new loss of taste or smell, weakness or fatigue, diarrhea, nausea and/or vomiting, and new skin rash.

Historical PDI data collected from the American Red Cross prior to the COVID-19 pandemic were used to detect trends in seasonal patterns of respiratory infections over time. Weekly PDI case counts were plotted and cross-referenced with public health data on pneumonia-, influenza-, and COVID-19–related mortality for the same period using publicly available data (https://www.cdc.gov/flu/weekly/index.htm). A centralized study database was developed to collect information provided on the enhanced PDI data form, including donor demographics, self-reported diagnosis, positive test results, and symptoms. The database also included SARS-CoV-2 RNA and antibody test results from the associated quarantined plasma units. Antibody test results from donations after the PDI donation were obtained by querying the blood centers’ operational databases.

### SARS-CoV-2 RNA and antibody detection.

Screening for SARS-CoV-2 RNA in plasma from PDI donors was performed using the Grifols Procleix SARS-CoV-2 TMA qualitative assay (50% LOD 2.46 copies/mL) on the Panther System in singlet. Initially RNA-reactive samples were retested in 11 replicates and considered RNA repeat reactive based on TMA reactivity in at least 1 of 11 replicates. If all 12 replicates were reactive, plasma was diluted 16-fold prior to testing 9 additional replicates. Viral load was estimated relative to dilutions (ranging from 1.25–56 gEq/mL) of heat-inactivated SARS-CoV-2, isolate USA-WA1/2020 (BEI resources NR-52286) tested in 12 replicates. Testing for anti–SARS-CoV-2 antibodies directed against SARS-CoV-2 proteins was performed using chemiluminescent immunoassays for anti-spike antibodies (Ortho VITROS Immunodiagnostic Products Anti-SARS-CoV-2 Total test) or anti-nucleocapsid antibodies (VITROS Immunodiagnostic Products Anti-SARS-CoV-2 Total N or Elecsys Anti-SARS-CoV-2).

### Viruses, cells, and in vitro infection assay.

Vero cells stably overexpressing TMPRSS2 (Vero-TMPRSS2) (provided courtesy of Stefan Pölhmann, Georg-August-University Göttingen, Germany) were cultured in DMEM supplemented with 10% FBS and 5 μg/mL blastidicin (InvivoGen). The human *ACE2* gene was cloned into lentiviral vector pLV-EF1a-IRES-Neo (Addgene) and lentivirus carrying the *ACE2* gene was made by transfecting HEK293T cells with pLV-EF1a-ACE2-IRES-Neo, psPAX2, and pVSV-G. To make Vero cells stably overexpressing both human ACE2 and TMPRSS2, Vero-TMPRSS2 cells were transduced with lentivirus carrying the *ACE2* gene and selected with G418 (MilliporeSigma). Stable Vero-ACE2-TMPRSS2 cells were cultured in DMEM supplemented with 10% FBS, 5 mg/mL blastidicin, and 500 mg/mL G418.

Susceptibility to SARS-CoV-2 infection was compared between Vero-TMPRSS2 and Vero-ACE2-TMPRSS2 cells. Vero-TMPRSS2 and Vero-ACE2-TMPRSS2 cells seeded in 96-well-plates were infected with serial dilutions of SARS-CoV-2 and incubated for 2 hours at 37°C followed by washing and culturing in fresh DMEM supplemented with 2% FBS. Cells were imaged 2–7 days after infection, and virus infection–induced CPEs were recorded. Five replicate wells were infected for each infection titer.

Vero-ACE2-TMPRSS2 cells were used to culture virus from SARS-CoV-2–positive human plasma. Vero-ACE2-TMPRSS2 cells seeded in 96-well plates were infected with undiluted and serial dilutions of SARS-CoV-2–positive plasma for 2 hours at 37°C, followed by washing and culturing in fresh DMEM supplemented with 2% FBS. Five replicate wells were infected for each infection titer. Three days after infection, cells were imaged to detect virus infection–induced CPE and culture supernatants were collected for RNA extraction. SARS-CoV-2 in culture supernatants was quantified by qRT-PCR.

### Mice.

B6.Cg-Tg(K18-ACE2)2Prlmn *Ifnar1^tm1.2Ees^*/J (K18-hACE2–Tg) and B6.129S2(Cg)-*Ace2^tm1(ACE2)Dwnt^*/J (hACE2-knockin) mice were purchased from The Jackson Laboratory and bred on-site at Vitalant Research Institute under barrier conditions in a specific pathogen–free vivarium. Both male and female mice were used and their age at time of infection ranged from 8 to 16 weeks.

The hACE2-knockin mice were given 1 mg/mouse InVivoPlus anti–mouse IFNAR-1 antibody (clone MAR1-5A3, Bio-X-Cell) i.p. 1 day prior to infection to increase susceptibility. The K18-hACE2–Tg mice were bred using K18-hACE2–Tg hemizygous females crossed with males without the transgene, producing litters that were a mixture of K18-ACE2–Tg hemizygotes and noncarriers all on an IFNAR1-KO B6 background. Noncarrier littermates were utilized for negative controls and anticoagulant toxicity studies.

Prior to infection, mice were relocated to our ABSL-3 facility. Intranasal infections and oropharyngeal swabs were done under inhaled anesthesia. Oropharyngeal swabs were taken using Puritan HydraFlock Flocked Swabs. Blood samples were collected into Sarstedt 20 μL Minivette POCT K3 EDTA tubes. Swab tips and blood samples were transferred into QIAzol (Qiagen) and stored at 4°C until RNA extraction. RNA was extracted from blood and swab samples using Qiagen miRNeasy Mini Kits. Mice were evaluated daily for visible symptoms of disease.

### Statistics.

Statistical analyses were performed using SAS version 9.4 (SAS Institute). All statistical tests were based on 2-tailed hypotheses. Differences were considered significant at a *P* value of less than 0.05. Comparisons of subgroups (i.e., RNAemic donors vs. non-RNAemic donors) were done using χ^2^ statistics for categorical responses (i.e., presence or absence of headache) and using *t*-test statistics for quantitative responses (i.e., number of symptoms). Wald 95% CIs were computed where applicable. In Figure 1, the plot of weekly PDI rate per 1,000 donations was smoothed using the default SAS quadratic Bézier spline interpolation of weekly rates (36).

### Study approval.

This study was approved by the Vanderbilt University Institutional Review Board (sIRB). Because blood centers already collect PDI as part of standard operating procedures, informed consent was not required. The National Heart, Lung, and Blood Institute (NHLBI) Recipient Epidemiology and Donor Evaluation Study-IV-Pediatric (REDS-IV-P) observational study monitoring board (OSMB) was responsible for oversight of data and safety for this study. The mouse experiments were performed with approval and oversight of the Institutional Animal Care and Use Committee at Labcorp Early Development Laboratories Inc. under Animal Welfare Assurance A3367-01.

## Author contributions

PS, RVF, SB, JJ, GS, MS, SHK, RPJ, CDG, and PJN planned experiments and analyzed data. MPB planned experiments. MOM, JJ, AC, AJH, RLT, JV, H Dave, DAK, and RPJ collected and analyzed data. BRS, DJW, and H Dawar analyzed data. TN, HK, SLS, and DEK provided clinical samples and data. PS, RPJ, and PJN wrote the manuscript. All authors reviewed and approved the manuscript.

## Supplementary Material

Supplemental data

## Figures and Tables

**Table 2:**
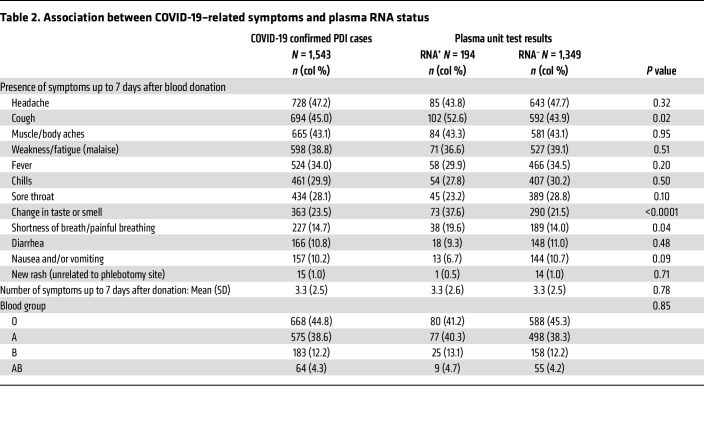
Association between COVID-19–related symptoms and plasma RNA status

**Table 1:**
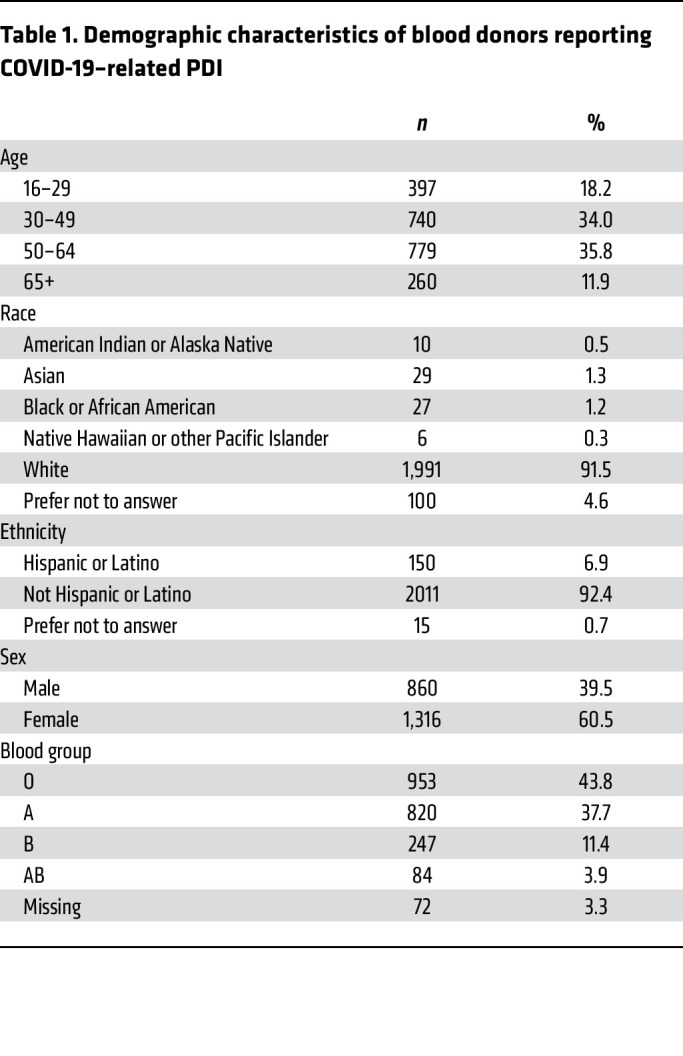
Demographic characteristics of blood donors reporting COVID-19–related PDI

## Version 1 07/14/2022

In-Press Preview

## Version 2 09/01/2022

Electronic publication
