# Supplementary material for: Frequent detection but lack of infectivity of SARS-CoV-2 RNA in presymptomatic, infected blood donor plasma
Source: J Clin Invest. 2022 Sep 1;132(17):e159876. doi: 10.1172/JCI159876 (PMC9435642; doi:10.1172/JCI159876)
Supplement: Supplemental data [file jci-132-159876-s160.pdf]

The NHLBI REDS Epidemiology, Surveillance and Preparedness of the Novel SARS-CoV-2 (RESPONSE) study is the responsibility of the following persons:

Vitalant Research Institute
M.P. Busch, P.J. Norris, and M. Stone, Vitalant Research Institute, San Francisco, CA

Data coordinating center
S.M. Mathew, Westat, Rockville, MD

Blood Collection Organizations
S. Stramer, American Red Cross (ARC), Gaithersburg, MD
D. Kessler, New York Blood Center (NYBC), New York, NY
B.A. Konkle, Blood Works Northwest, Seattle, WA
B. Custer, Vitalant Research Institute, San Francisco, CA

Publications Committee Chairman
P.M. Ness, Johns Hopkins University, Baltimore, MD

Steering Committee Chairpersons
S.H. Kleinman, University of British Columbia, Victoria, BC, Canada
C.D. Josephson, Emory University, Atlanta, GA

National Heart, Lung, and Blood Institute, National Institutes of Health
S.A. Glynn and K. Malkin

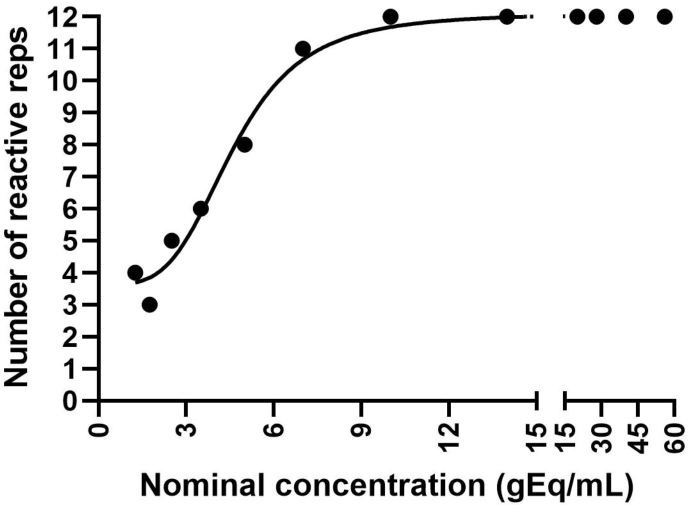

Figure S1. Standard curve for SARS-CoV-2 viral load estimation in plasma. Heat-inactivated SARS-CoV-2 virus (quantified in gEq/mL) was serially diluted to 12 concentrations ranging from 1.25 to 56 gEq/mL and tested in 12 replicates using a qualitative TMA assay for detection of SARS-CoV-2 RNA. A best fit curve was determined using a four-parameter logistic regression model.

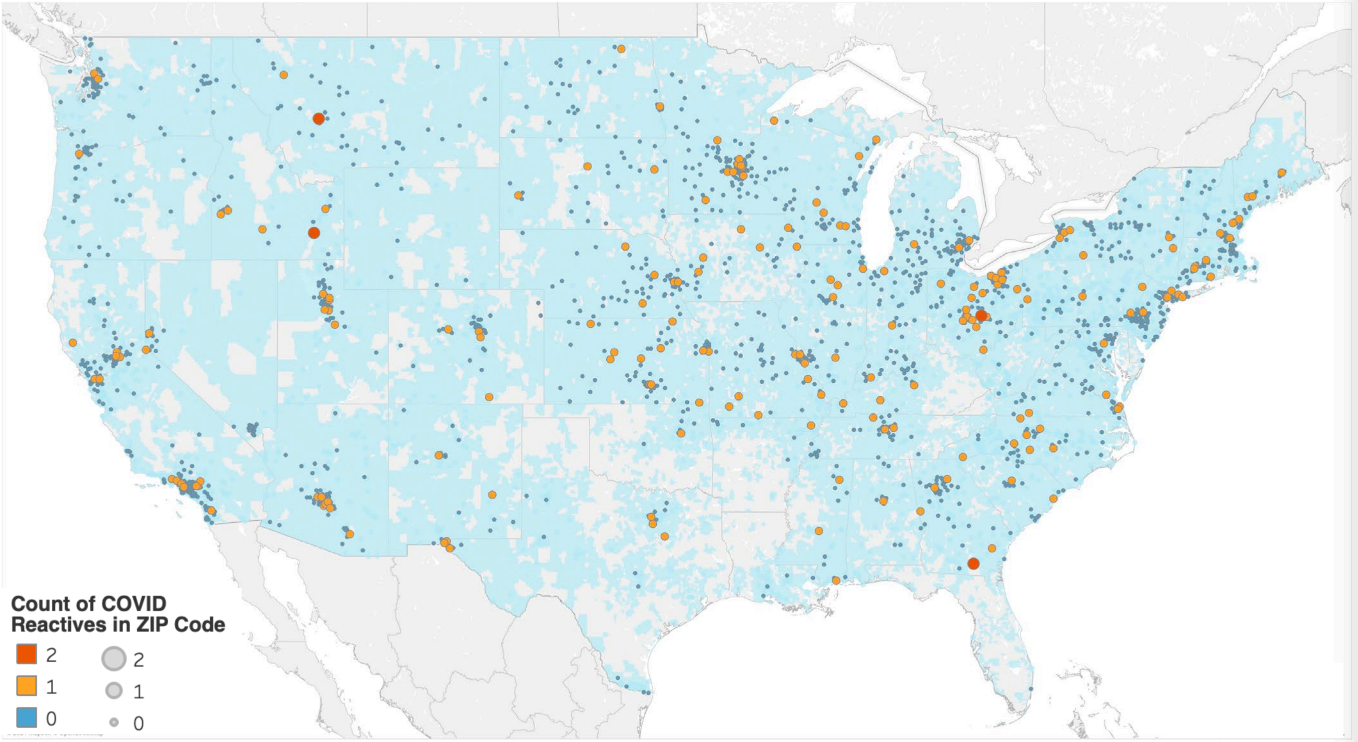

Figure S2. Geographical distribution of PDI reports and TMA reactive units. The map displays in light blue shading zip codes where in the continental US blood units were collected by the participating blood centers during the study. The dots represent PDI donor donation locations by zip code, with the size of the dot proportional to the number of PDI donors. PDI donors without detectable SARS-CoV-2 RNA are dark blue, and dots with one or two PDI donors with detectable SARS-CoV-2 RNA are amber or red, respectively.

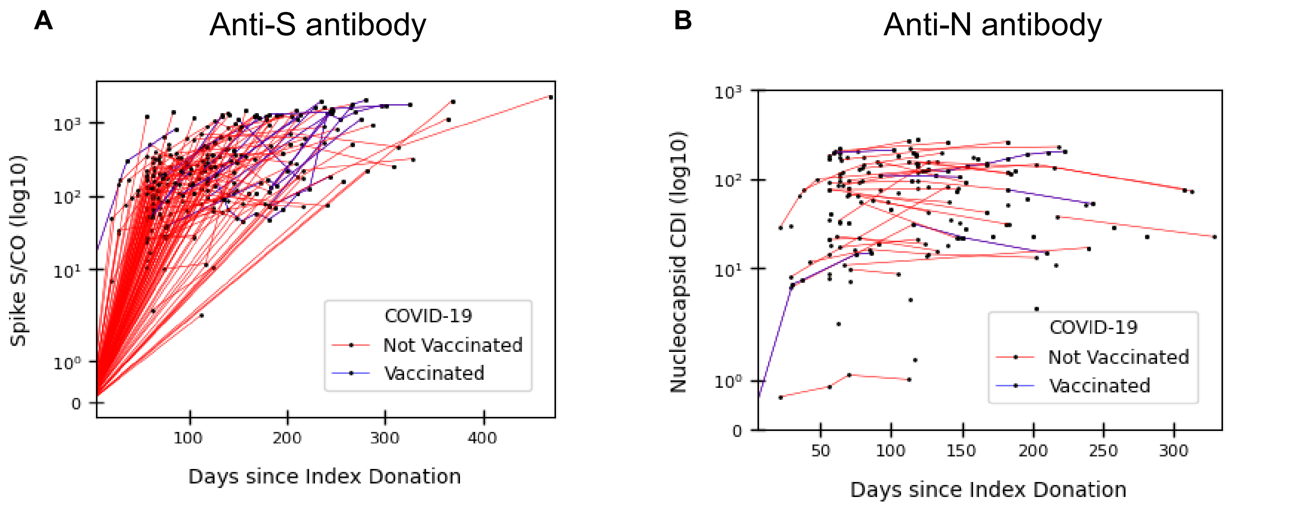

Figure S3. Antibody evolution in PDI and general blood donors. Universal blood donor screening for anti-S antibodies was performed, and positive samples were reflexively tested for anti-N antibodies. Data from PDI donors' subsequent donations were tested for (A) anti-S antibodies (n=132 donors) and (B) anti-N antibodies (n=106 donors).

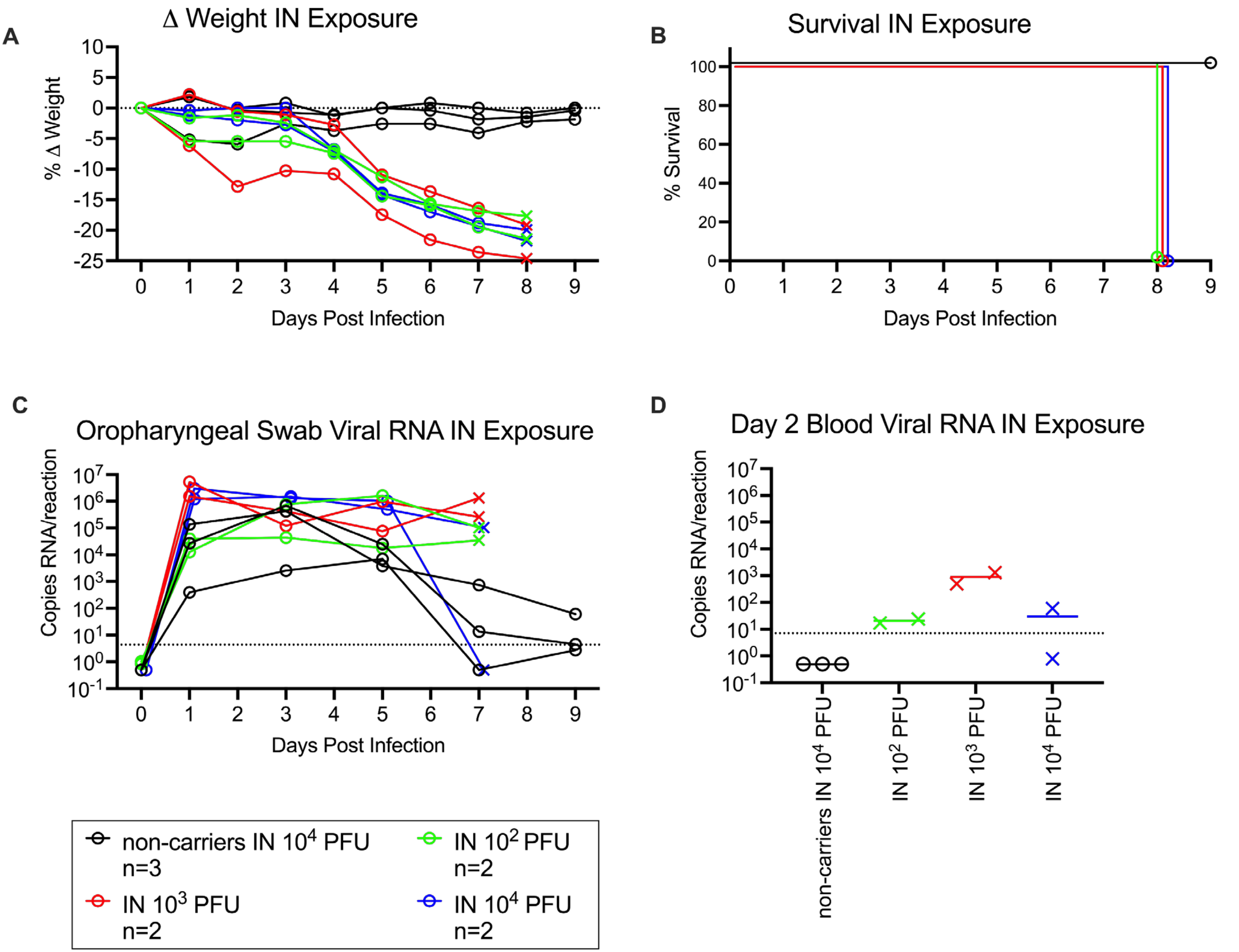

Figure S4. Establishment of K18-hACE2 IFNAR infection model. K18-hACE2 IFNAR KO mice or IFNAR KO littermates that do not carry the K18-hACE gene (non-carriers) were infected intranasally with 1.1 x 10^2 PFU, 1.1 x 10^3 PFU, or 1.1 x 10^4 PFU of the B1.1.7 variant of SARS-CoV2. (**A**) Weights were measured daily, and the % weight change is plotted for each mouse over time. (**B**) Survival over time is plotted with all non-carrier controls surviving up until the end of the experiment at day 9, and all K18-hACE2+ mice at all doses dying at day 8. (**C**) Oropharyngeal swabs were taken at days 0, 1, 3, 5, 7, and 9, RNA was isolated, and SARS-CoV2 RNA levels were measured by qRT-PCR. Values are plotted for each mouse. (**D**) On Day 2, 20 µL of EDTA whole blood was collected, RNA was isolated, and SARS-CoV2 RNA levels were measured by qRT-PCR. Values are plotted for each mouse. X indicates non-surviving mouse. Dashed line indicates max value detected among 63 negative blood sample controls or 38 negative swab sample controls plus 0.5 and is used as a cutoff for positive signal. When no viral RNA was detected, a value of 0.5 was assigned.

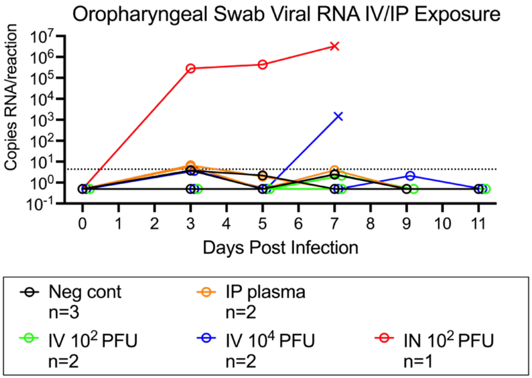

Figure S5. Oropharyngeal RNA not detected following intravenous exposure of K18-hACE2 IFNAR mice to SARS-CoV-2 B1.1.7. K18-hACE2 IFNAR KO mice were given either 1.1 x 10^2 PFU or 1.1 x 10^4 PFU B1.1.7 intravenously, 1.1 x 10^2 PFU B1.1.7 intranasally (positive control), or 500 µL SARS-CoV2 RNA+ human plasma intraperitoneally. IFNAR KO littermates that do not carry the K18-hACE gene (non-carriers) with no exposure were included as negative controls. Oropharyngeal swabs were taken at days 0, 3, 5, 7, 9, and 11, RNA was isolated, and SARS-CoV2 RNA levels were measured by qRT-PCR. Values are plotted for each mouse. X indicates non-surviving mouse. Dashed line indicates max value detected among 38 negative swab sample controls plus 0.5 and is used as a cutoff for positive signal. When no viral RNA was detected, a value of 0.5 was assigned.

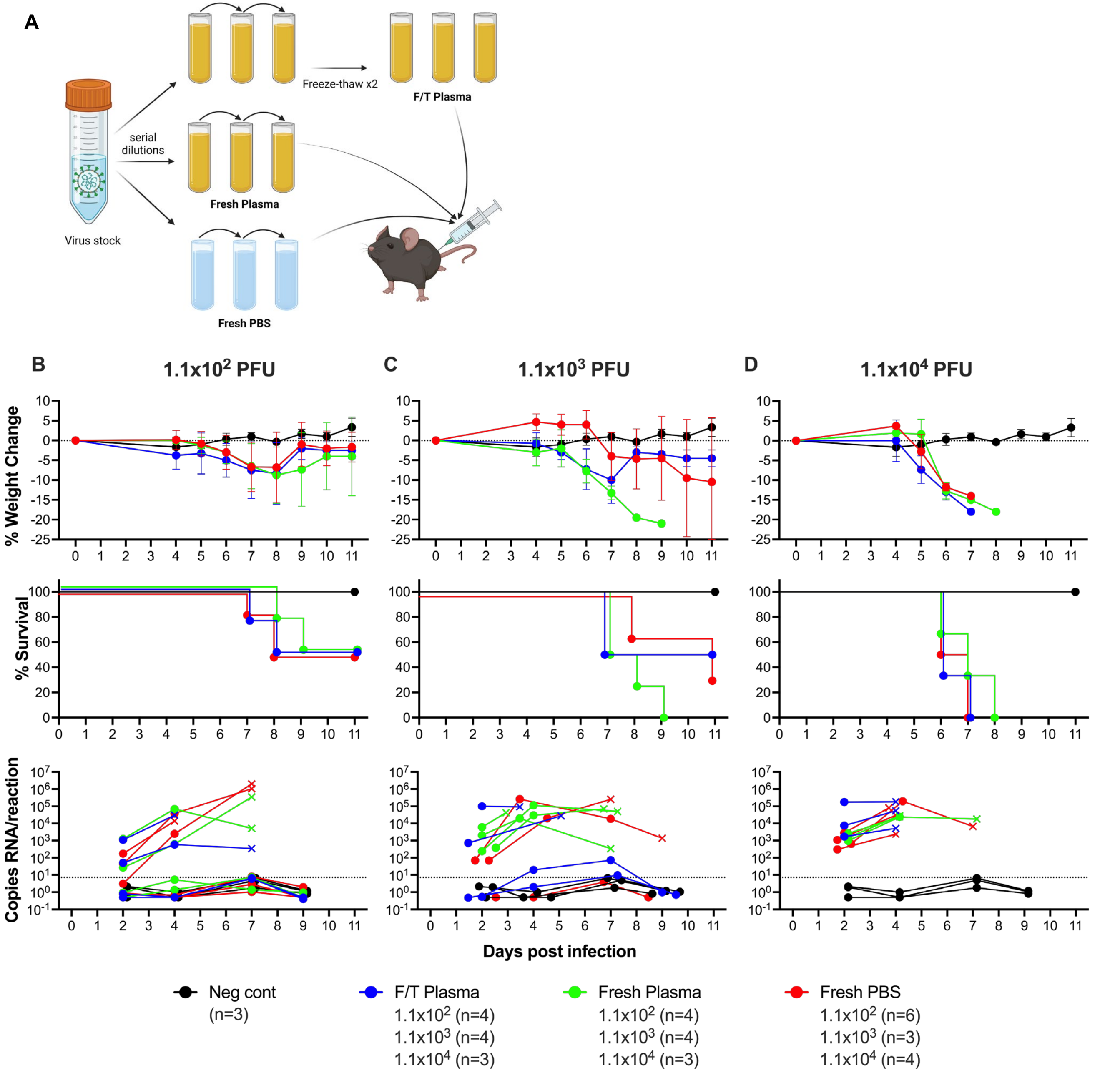
A
Virus stock
serial dilutions
Freeze-thaw x2
F/T Plasma
Fresh Plasma
Fresh PBS
B
1.1x10^2 PFU
C
1.1x10^3 PFU
D
1.1x10^4 PFU
% Weight Change
% Survival
Copies RNA/reaction
Days post infection
Neg cont
(n=3)
F/T Plasma
1.1x10^2 (n=4)
1.1x10^3 (n=4)
1.1x10^4 (n=3)
Fresh Plasma
1.1x10^2 (n=4)
1.1x10^3 (n=4)
1.1x10^4 (n=3)
Fresh PBS
1.1x10^2 (n=6)
1.1x10^3 (n=3)
1.1x10^4 (n=4)

Figure S6. Plasma and freeze/thaw of samples do not alter infectivity of SARS-CoV2 in our model system. (A) Aliquots of B1.1.7 SARS-CoV2 and of a SARS-CoV2 RNA$^+$ human plasma sample were thawed, and dilutions virus were prepared in the plasma. These diluted viral stocks were frozen at -80ºC for 2 days, thawed, then frozen again for 4 days and thawed on infection day for use (F/T Plasma). Second aliquots of the same lot of virus and plasma sample were thawed on infection day and fresh dilutions were made in the plasma (Fresh Plasma) and PBS (Fresh PBS). K18-hACE2 IFNAR KO mice were given either (B) 1.1 x 10^2 PFU, (C) 1.1 x 10^3 PFU, or (D) 1.1 x 10^4 PFU B1.1.7 intraperitoneally. IFNAR KO littermates that do not carry the K18-hACE gene (non-carriers) with no exposure were included as negative controls and are shown on each dose plot for comparison. Weights were measured daily, and the % weight change was calculated for each mouse over time with mean change in weights and standard deviation plotted for each group. % Survival over time is plotted by group. At indicated time-points, 20µL of EDTA whole blood was collected, RNA was isolated, and SARS-CoV2 RNA levels were measured by qRT-PCR. Three new mice in the 1.1x10^2 PFU Fresh PBS group were run with this experiment, remaining data from the Fresh PBS groups were included from Fig.6 and are included here to facilitate comparisons. Values are plotted for each mouse. Dashed line indicates max value detected among 63 negative blood sample controls plus 0.5 and is used as a cutoff for positive signal. When no viral RNA was detected, a value of 0.5 was assigned.

Table S1. Enhanced PDI data collection for participating blood collection organizations

	ARC	NYBC	Vitalant
Enhanced PDI data collection period	Jan 2020 – July 2021	Sept 2020 – July 2021	Apr 2020 – July 2021
# of PDI reports collected	1,715	32	429
Days between donation and PDI report Mean (SD)	4.1 (2.6)	5.4 (3.0)	5.7 (3.8)

ARC: American Red Cross, NYBC: New York Blood Center
